# Cultural, Religious, and Spiritual Influences on Communication in Pediatric Palliative Care: A Narrative Review Focused on Children with Severe Neurological Conditions

**DOI:** 10.3390/children12081033

**Published:** 2025-08-06

**Authors:** Francesca Benedetti, Luca Giacomelli, Simonetta Papa, Viviana Verzeletti, Caterina Agosto

**Affiliations:** 1 Department of Women’s and Children’s Health, University of Padova, 35128 Padua, Italy; francesca.benedetti@aopd.veneto.it (F.B.); caterina.agosto@aopd.veneto.it (C.A.); 2 Polistudium SRL, 20121 Milan, Italy; simonetta.papa@polistudium.it (S.P.); viviana.verzeletti@gmail.com (V.V.)

**Keywords:** advance care planning, cultural humility, interdisciplinary care, neuropalliative care, pediatric palliative care, prognostic communication, spirituality

## Abstract

Pediatric palliative care (PPC) aims to enhance the quality of life of children with life-limiting conditions and their families through individualized, interdisciplinary support. Among this population, children with neurological diseases represent a substantial and growing group, often facing prolonged disease courses, cognitive impairment, and high prognostic uncertainty. Effective communication is central to PPC; however, it remains deeply influenced by cultural, religious, and spiritual frameworks that shape family perceptions of illness, suffering, and decision-making. This narrative review explores communication strategies in PPC, with a specific focus on children with neurological conditions, highlighting conceptual foundations, cross-cultural variations, and emerging best practices. Key findings highlight the importance of culturally humble approaches, family-centered communication models, and structured tools, such as co-designed advance care planning and dignity therapy, to enhance communication. Additionally, the review highlights the presence of ethical and interdisciplinary challenges, particularly in neonatal and neurology settings, where misaligned team messaging and institutional hesitancy may compromise trust and timely referral to palliative care. Future research, policy, and clinical education priorities should advocate for models that are inclusive, ethically grounded, and tailored to the unique trajectories of neurologically ill children. Integrating cultural competence, team alignment, and family voices is essential for delivering equitable and compassionate PPC across diverse care settings.

## 1. Introduction

Pediatric palliative care (PPC) is a holistic, interdisciplinary approach aimed at improving the quality of life of children with life-limiting or life-threatening conditions and their families through comprehensive physical, emotional, psychosocial, and spiritual support [1]. Among the pediatric population requiring PPC, a substantial proportion is affected by non-oncological diseases, particularly serious neurological conditions, such as congenital brain disorders, progressive neurodegenerative diseases, and severe acquired brain injuries [1,2]. These patients often experience prolonged, dynamic trajectories of care characterized by complex clinical needs, cognitive and communicative impairments, and significant prognostic uncertainty [3].

In response to these specific challenges, the emerging discipline of neuropalliative care has evolved. Neuropalliative care focuses on the holistic needs of children with serious neurological illnesses, encompassing symptom management, quality of life optimization, anticipatory decision-making support, and goal-concordant care throughout the disease course [4]. The Consensus Statement from the International Neuropalliative Care Society underscores the critical need to develop tailored communication frameworks and care delivery models specifically adapted to the unique trajectories and needs of children with neurologic conditions [4].

Communication in PPC must recognize the profound impact of cultural, religious, and spiritual belief systems on families’ understanding of illness, suffering, and medical decision-making [5,6]. Such beliefs significantly shape perspectives on prognosis disclosure, perceived suffering, end-of-life decision-making roles, and quality of life [7]. Particularly in multicultural societies, culturally humble communication—characterized by respect, open inquiry, and genuine curiosity about families’ worldviews—is essential for fostering trust and therapeutic alliances [5,8]. Importantly, spirituality should not be regarded merely as a religious phenomenon, but rather as an integral dimension of meaning-making and coping for families navigating serious pediatric illnesses [7,9]. Notably, for the purposes of this review, we differentiate between religion (a structured system of shared beliefs, practices, and rituals often organized within a community) and spirituality (a broader, often personal search for meaning, connection, and transcendence, which may or may not be connected to formal religion) [10]. Recent evidence increasingly emphasizes that PPC delivery must be responsive to the cultural and existential values of all families, irrespective of traditional notions of minority status [11,12]. Disparities in palliative care have been documented even in well-resourced settings, reflecting the urgent need for communication practices that adapt to the diversity of beliefs and experiences of pediatric patients and their families [13,14].

This review aims to synthesize available evidence on communication strategies in pediatric palliative and neuropalliative care, with particular attention to cultural, spiritual, and religious considerations. We explore the conceptual foundations, cross-cultural variations, ethical issues, and emerging best practices, with the ultimate goal of supporting the development of culturally inclusive and individualized communication models for children with life-limiting neurological conditions and their families.

## 2. Methods

A narrative review approach was adopted to synthesize the current evidence regarding communication practices in PPC, with particular attention to children with severe neurological conditions and the influence of cultural, religious, and spiritual factors.

The literature search was finalized in April 2025 and conducted across major biomedical databases, including PubMed, Embase, Scopus, and CINAHL. Manual searches of references from key articles and guidelines were also performed to ensure completeness. The following search string was used as the primary query across the databases:

((“complex”) AND (pediatric* OR paediatric* OR child*) AND (palliative OR terminal OR supportive) AND (spirit* OR religio* OR cultur*)).

Searches were restricted to articles published in English without time constraints. Original research studies (qualitative, quantitative, and mixed methods) and review articles were considered eligible if they explored aspects of communication, cultural diversity, spirituality, religion, or decision-making in pediatric palliative care contexts. Particular emphasis was placed on studies involving children with complex chronic conditions, especially neurological diseases, given their prevalence in PPC populations.

Additionally, grey literature, such as consensus statements, professional guidelines, and conference proceedings, was screened to capture emerging best practices. Articles focusing exclusively on adult palliative care populations unrelated to communication or cultural diversity issues were excluded.

A total of 170 articles were identified through the initial search. After screening and full-text review, 55 articles were included in the final synthesis (Table 1).

After screening titles and abstracts for relevance, the full texts of potentially eligible sources were reviewed in detail. Data from the included publications were extracted and organized into thematic categories reflecting the main domains of interest: communication practices in PPC, cultural and religious influences on communication and decision-making, and considerations specific to children with severe neurological conditions. The findings were synthesized narratively and descriptively, highlighting key themes, recurring challenges, and illustrative examples from the literature. Where appropriate, we prioritized higher-level evidence and more recent studies while acknowledging the contribution of earlier landmark works. To reflect the most contemporary understanding, priority was generally given to publications from 2010 onward, although landmark papers prior to this date were included if they were deemed foundational.

Given the narrative review design, the findings are presented as an integrated thematic synthesis without a separate “Results” section.

## 3. Conceptual Foundations of Cross-Cultural Communication in Pediatric Palliative Care

Effective communication lies at the heart of PPC, ensuring that families’ values, beliefs, and preferences are honored, alongside medical goals. In cross-cultural settings, communication must address not only informational needs but also the diverse spiritual, religious, cultural, and social frameworks that shape families’ experiences of illness, suffering, and decision-making [6,15].

Culture, defined as the learned and shared patterns of behavior, beliefs, and values within groups, profoundly impacts health perceptions and healthcare interactions. In PPC, culture influences every stage of care, from diagnosis to end-of-life decisions and bereavement practices. Recognizing this, culturally humble communication—emphasizing openness, respect, and genuine curiosity toward others’ worldviews—has become a foundational principle [6,16].

However, communication across cultures often encounters such barriers. Language differences, varying concepts of illness and death, differing expectations of truth-telling, and assumptions about decision-making authority can cause tension. Healthcare providers may unintentionally impose their own cultural norms, reinforcing systemic inequities. Fear of causing offense and lack of formal training in cultural humility exacerbate these challenges. Moreover, families’ understanding of palliative care itself may be shaped by sociocultural narratives that differ significantly from Western medical models [5,17,18,19].

Spirituality and religion are essential dimensions. As highlighted by Davies et al. [20], addressing children’s spiritual needs is a core component of total care, yet it often remains underexplored. Parents draw on a wide range of religious, spiritual, and philosophical beliefs when facing their child’s illness. These beliefs may influence their acceptance of prognosis, interpretation of suffering, and views on medical interventions. Parents often experience complex tensions between their spiritual or religious beliefs and the medical realities of their child’s condition. These tensions arise when faith-based values—such as beliefs in miracles or divine will—conflict with medical recommendations that prioritize the child’s comfort and minimize suffering. On the one hand, spirituality and religion may provide parents with meaning, hope, and emotional resilience during the decision-making process. On the other hand, they can also lead to internal conflict and decisional distress, particularly when accepting palliative or end-of-life care feels incongruent with their religious convictions [6].

Importantly, culturally sensitive communication must avoid simplistic categorization. Families are not monolithic, and intra-group variation can be considerable. Attention must be paid to intersectionality, recognizing that cultural identity interacts with factors such as socioeconomic status, education level, migration history, and prior healthcare experiences [21]. As emphasized by Rosenberg et al. [12], building trust requires acknowledging these complex layers, addressing systemic power imbalances, and fostering individualized and respectful dialogue.

Lastly, a growing body of literature stresses that cultural humility is not a fixed competence but an ongoing process of self-reflection, learning, and relationship building. This requires practitioners to engage families openly, acknowledge uncertainty, and adapt communication styles to support authentic, goal-concordant care across diverse cultural landscapes [5].

## 4. Cultural Differences Shaping Communication in Pediatric Palliative Care

Communication practices in PPC are significantly shaped by cultural, linguistic, spiritual, religious, and systemic factors. Cultural, religious, and linguistic dimensions interact in nuanced ways, shaping each family’s expectations and responses to care (Table 2) [5,9,20,21,22,23,24,25,26,27].

However, it must be recognized that the vast majority of available evidence stems predominantly from oncological contexts, with fewer studies focusing specifically on children with neurological conditions. The extrapolation to broader PPC populations must be undertaken cautiously, with attention to disease-specific and culturally nuanced realities.

### 4.1. Language and Interpretation Challenges

Language barriers remain one of the most consistent obstacles to effective communication in PPC. Professional interpreters are underutilized, with interpreter services documented in fewer than 5% of critical conversations with culturally diverse families [26]. Dreier et al. [25] found that limited language proficiency significantly hampers prognostic understanding and increases emotional distress in families.

Informal interpretation by family members can introduce inaccuracies and emotional strain [5,21]. In addition, misunderstandings are compounded by cultural variations in non-verbal communication and relational hierarchies [17]. Monette [16] and Upshaw et al. [28] stressed the importance of integrating professional interpretations with culturally sensitive communication strategies. Addressing these barriers requires not only access to interpreters but also clinician training in culturally responsive engagement [29].

### 4.2. Truth-Telling, Prognosis Disclosure, and Decision-Making

Truth-telling and prognosis-disclosure practices vary widely across cultures. In Western medical ethics, direct and transparent disclosures are prioritized. However, many non-Western cultural frameworks endorse protective non-disclosure as a compassionate practice [15,22].

Peng et al. [23] reported that in Taiwan, families often prefer not to disclose terminal diagnoses to children, reflecting Confucian values of familial harmony. Lin et al. [24] further discussed that Chinese cultural norms discourage explicit discussions about death, often leading to misinterpretations of medical information. Glyn-Blanco et al. [30] similarly emphasized that cultural beliefs about death and dying significantly shape family decisions regarding information sharing.

Thorvilson et al. [15] demonstrated how facilitating culturally congruent palliative transport enabled families to incorporate traditional rituals into the dying process, illustrating how cultural norms influence both communication and end-of-life care preferences.

### 4.3. Religion, Spirituality, and Cultural Contexts

Spirituality and religion are distinct but interrelated aspects that often influence end-of-life decision-making for many families; however, wide diversity exists within and across faith traditions. Puchalski [27] emphasized that although spirituality has been increasingly recognized, research remains limited and needs to be strengthened to support evidence-based spiritual care practices.

Pereira-Salgado et al. [31] showed that religious leaders across multiple faiths view advance care planning (ACP) favorably once adequately understood, but stressed the necessity of avoiding assumptions based on religious affiliation alone. Families’ attitudes towards end-of-life care reflect the intersecting influences of personal religiosity, cultural traditions, family dynamics, and individual beliefs.

Al Mutair et al. [19] illustrated that among Muslim families, interpretations of divine will vary, affecting preferences for life-sustaining interventions. Similarly, Wiener et al. [32] found that adolescents and young adults experience varying degrees of stress related to discussing spirituality during ACP, underscoring the need for individualized approaches.

### 4.4. Broader Systemic and Structural Barriers

In addition to interpersonal communication challenges, systemic inequities and structural barriers also impact PPC delivery. Islam et al. [5] and Rosa et al. [33] emphasized that minority families often experience mistrust of healthcare systems, feelings of marginalization, and a lack of culturally congruent care options.

Institutional inflexibility, lack of cultural humility training, and poor adaptation of palliative care frameworks to diverse needs have been recurrently identified as barriers [17,30]. Upshaw et al. [28] stressed that cultural, developmental, and support structure considerations must be integral to PPC and ACP for adolescents and young adults, who navigate the complex intersections of autonomy, family involvement, and cultural values.

Efforts to foster cultural safety—through organizational change, clinician education, and systematic accommodation of diverse needs—are essential to improve equitable communication and care in PPC [5,9,21,22,23,24,25]. These cultural, religious, and linguistic dimensions interact in nuanced ways, shaping each family’s expectations and responses to the care of their dying relatives. A summary of the key cross-cultural variables and their implications for PPC communication is provided in Table 2.

## 5. Communication in Neurologically Ill Children: Special Challenges

Communication in the care of neurologically ill children is uniquely shaped by prognostic ambiguity, developmental limitations, interdisciplinary fragmentation, and ethical complexities. These challenges require structured, longitudinal, and ethically responsive models of care (Table 3) [4,11,25,34,35,36,37,38].

In this section, we address communication challenges in both neonatal intensive care unit (NICU) settings and children with progressive neurological conditions under a unified heading. This choice reflects the significant clinical overlap between these populations, as the majority of severe neurological conditions manifest during the neonatal period or result from perinatal complications. In addition to prematurity-related brain injury, many severe neurological disorders in children, including those requiring palliative care, have genetic or congenital origins that become evident in the NICU setting or the first months of life. Therefore, the NICU often represents the first point of care where families and clinicians face communication challenges associated with these complex, life-limiting conditions.

### 5.1. Prognostic Uncertainty and Evolving Dialogue

Children with serious neurological illnesses often experience prolonged and complex trajectories marked by diagnostic uncertainty, variable clinical progression, and profound developmental challenges [4].

Prognostication under these conditions is inherently uncertain and evolves over time. The traditional focus on life expectancy is insufficient; clinicians must also explore dimensions such as functional potential, comfort, communication capacity, and quality of life. Bergstraesser et al. emphasized that discussions around prognosis should move beyond “how long” toward “what will life look like,” integrating families’ values and long-term hopes for their children [34].

This approach aligns with the principles of parallel planning—simultaneously preparing for both the best and worst outcomes—particularly valuable in perinatal and neonatal settings, where outcome trajectories are difficult to predict [11,34]. Such nuanced discussions benefit from early iterative engagement that validates uncertainty and fosters a collaborative therapeutic alliance between the care team and the family [4].

### 5.2. Enhancing Family Engagement Through Structured Models

Given that many neurologically affected children lack the capacity for direct participation in care decisions, effective family engagement is paramount. Structured communication models like the Pediatric Serious Illness Communication Program (PediSICP) have demonstrated feasibility and benefits in this context. DeCourcey et al. found that PediSICP facilitated more goal-aligned care, improved therapeutic alliance, and reduced parental anxiety—even in scenarios marked by clinical ambiguity, such as neurodevelopmental conditions [35].

Such models encourage the co-creation of care plans grounded in what matters most to families and support shared decision-making, even in the face of prognostic uncertainty. They enable anticipatory guidance while respecting families’ coping rhythms and cognitive readiness [4,22].

These findings echo the need, underscored in the International Neuropalliative Care Society’s consensus, for specialized tools and training that support communication tailored to neuro-complex populations [4]. Such structured approaches promote alignment between medical goals and family priorities across diverse cultural and clinical settings [4,22].

### 5.3. Communication Models in NICU and Progressive Neurological Conditions

In neonatal and early childhood settings, communication challenges are further intensified by time-sensitive decisions, fragmented care structures, and emotional distress. Rent et al. noted that the NICU environment often includes multiple rotating teams, which can lead to inconsistent messaging and parental confusion [11,25]. Addressing this requires deliberate coordination and continuity, ideally through family-centered team meetings and shared care plans.

Hrdličková et al. demonstrated that including the primary treatment team in initial palliative consultations significantly enhances parental trust, improves interdisciplinary coherence, and provides critical psychosocial insights [36]. Their study also highlighted the novel practice of inviting parental feedback on written summaries of these meetings—an innovation that fosters family empowerment and narrative integrity.

Communication in progressive neurological conditions should be framed as a longitudinal process. Lau et al. called for the development of adaptable models that evolve with the child’s condition, allowing care teams to revisit and reframe goals across disease stages [4]. Importantly, the documentation of decisions and values over time can bridge transitions between inpatient, outpatient, and home-based care settings, facilitating consistency and preparedness [4].

Spiritual and cultural beliefs profoundly shape families’ perceptions of disability, suffering, and end-of-life choices. As highlighted by Wiener et al., culturally humble communication should avoid prescriptive assumptions and prioritize open inquiry into each family’s worldview [22]. This is particularly important when discussing interventions such as tracheostomy or long-term mechanical ventilation, which may evoke existential or moral considerations beyond the biomedical risk-benefit analysis [11,37].

## 6. Innovations and Best Practices in Cross-Cultural Pediatric Palliative Communication

Communication strategies in PPC must continually evolve to meet the complex needs of children and their families from diverse cultural backgrounds. Recent innovations have focused on promoting cultural sensitivity, improving accessibility, and fostering family-centered and ethically grounded models [8,35,36,39,40]. A comparative overview of recent innovations, including co-designed ACP tools, digital communication platforms, and structured communication protocols, is provided in Table 4, with a focus on their applicability to neuropalliative care contexts.

An important advancement is the increasing use of co-designed tools for ACP among culturally and linguistically diverse (CALD) populations. Chauhan et al. [39] highlighted that direct translation of ACP resources often fails to capture the nuanced cultural and religious variations that influence end-of-life preferences. Their iCanCarePlan study emphasizes the importance of culturally adapted ACP materials and co-design approaches that involve stakeholders from CALD backgrounds in creating communication tools rather than merely translating existing frameworks. This method respects diverse conceptualizations of illness, autonomy, and family roles in decision-making.

Similarly, Burke et al. [13] emphasized the need to enhance healthcare practitioners’ cultural competence. Their systematic review demonstrated that communication challenges in palliative care frequently stem from language difficulties, fear of cultural insensitivity, and differing expectations regarding truth-telling and family involvement. They advocated for individualized communication strategies, continuous cultural humility education, and institutional support for culturally responsive care.

Innovative frameworks have incorporated family centered dignity therapy to strengthen relational bonds at the end of life. Lin et al. [8] developed a pediatric family-based dignity therapy protocol specifically adapted for Chinese cultural contexts. By involving families in structured, meaningful conversations and preserving these memories, the intervention supports psychological resilience and acknowledges the centrality of relational and spiritual dynamics in many non-Western cultures.

Digital communication platforms have also emerged as adjuncts to traditional in-person interactions. Rosa et al. [40] explored the use of WhatsApp messaging between PPC psychologists and adolescents with life-limiting illnesses. While WhatsApp enables more immediate patient-centered interactions, symptom monitoring, and emotional support, it is important to note that this approach has not been formally evaluated in neuropalliative care populations, including parents and children with neurodevelopmental disabilities. The potential role of such tools remains promising but requires further validation.

Most of these innovations have been piloted in oncology-based palliative care settings [13,40]. Applying them to children with neurologic illness—who often present with earlier onset, slower disease progression, and limited communication capacity—requires careful adaptation and empirical validation. Structured interventions must account for the complexities of neurological prognosis, decision-making capacity, and long-term family involvement [4].

Innovative communication strategies must also address the ethical and interdisciplinary challenges that frequently arise in the care of children with neurological diseases. These cases are often marked by prognostic uncertainty, moral complexity, and fragmented care structures, requiring more than just clinical expertise. A central ethical dilemma in neuropalliative care involves decisions regarding the initiation, withholding, or withdrawal of life-sustaining treatment [37,38]. As Meglič et al. [38] observed, divergent medical opinions among neurologists, intensivists, and palliative care providers can result in inconsistent messages to families and delayed palliative care integration. In some cases, children may be excluded from PPC services, not based on prognosis, but due to unresolved team conflict or a lack of institutional consensus [38]. These scenarios pose serious ethical concerns and illustrate the importance of coordinated, ethically attuned communication. In high-acuity settings, such as NICUs and PICUs, these tensions are often amplified. Sorin et al. [38] emphasized that progressive, emotionally sensitive communication is essential to build parental trust and facilitate ethical decision-making. Families benefit when complex issues are introduced gradually in emotionally safe environments and when clinicians avoid abrupt, fragmented, or overly technical discussions [37].

To address this issue, ethically informed communication models should integrate the primary treatment team into early palliative care discussions and ensure consistency in messaging across disciplines. Hrdličková et al. [36] found that families highly valued the presence of trusted clinicians during PPC introductions and appreciated being invited to review and edit written summaries of these meetings. This practice promotes transparency and narrative inclusivity.

Structured ACP programs, such as PediSICP, have also demonstrated potential in promoting goal-aligned, ethically sound decision-making, even in the presence of diagnostic ambiguity [35]. However, their use in neuropalliative care must be adapted to the layered emotional, developmental, and moral complexities of this population.

Ultimately, ethically grounded communication is relational and systemic. Shared ethical language, interdisciplinary training, and institutional commitment are required to reduce communication silos and moral distress. Ensuring ethically sound, culturally humble, and interdisciplinary communication models is essential for delivering neuropalliative care that respects the dignity, values, and humanity of every child and family [21].

## 7. Discussion

The delivery of high-quality pediatric palliative and neuropalliative care requires culturally humble, individualized communication approaches that respect families’ diverse worldviews, spiritual needs, and decision-making frameworks. This review underscores that children with neurological conditions—who represent a substantial proportion of PPC recipients—face distinct challenges arising from prolonged disease trajectories, developmental limitations, and enduring prognostic uncertainty.

While there is growing awareness of the role of cultural, religious, and spiritual beliefs in shaping communication preferences, most available evidence continues to originate from oncology-based PPC models. Therefore, dedicated multi-centered research exploring communication strategies tailored to neurologically ill children is urgently needed, especially in cross-cultural and multidisciplinary settings. Future work should investigate how structured tools, such as culturally co-designed ACP models and dignity therapies, can be ethically and developmentally adapted for children with neurocomplex conditions.

At the systemic level, communication frameworks must address the ethical and interdisciplinary tensions that can arise when care teams hold divergent views about prognosis or treatment goals. As highlighted in recent studies, delayed or misaligned messaging, particularly in the NICU and neurology contexts, may inadvertently exclude children from timely PPC referral and erode family trust. Shared decision-making models, structured documentation, and consistent inclusion of the treating team in early PPC conversations are promising practices that merit wider implementation and evaluation.

From an institutional and policy perspective, equity, diversity, and inclusion should become foundational principles—not only in research design but also in workforce training, guideline development, and routine clinical practice. Communication strategies must embrace the intersectionality of family experiences and avoid oversimplified assumptions based on religious or ethnic labels. Families must be approached as experts in their own values and goals, and providers must be supported in cultivating skills that center on relational, context-aware, and ethically grounded communication. Clinicians are thus encouraged to explore individual family preferences regarding disclosure early in the care trajectory, utilizing flexible and culturally sensitive models of communication that balance transparency with respect for family values. Moreover, they should maintain openness, avoid stereotyping based on apparent religious identity, and integrate the exploration of spiritual needs into routine palliative care discussions.

Moving forward, efforts to improve PPC communication must prioritize the validation of tailored models across neurological conditions, the integration of ethics-informed practices, and the development of tools that are flexible across cultural and developmental dimensions. These steps are essential not only for clinical excellence but also for the moral integrity of care itself. Supporting the dignity, agency, and humanity of every child and family—regardless of cultural background or neurological status—must remain at the heart of palliative care.

### Study Limitations

This narrative review has several limitations that should be acknowledged. As a narrative rather than a systematic review, the selection and synthesis of the literature were not guided by formal quality appraisal tools or standardized inclusion criteria, which may introduce selection bias. Moreover, thematic synthesis relies on the authors’ interpretation of heterogeneous sources, potentially contributing to subjectivity in how themes were identified and weighted. The diversity of study designs, populations, and cultural contexts further limits the generalizability of these findings.

## 8. Conclusions

Effective communication in pediatric palliative and neuropalliative care must be individualized, culturally humble, and aligned with families’ values and priorities. This review shows that communication in these settings is profoundly shaped by intersecting cultural, religious, and spiritual beliefs and systemic factors such as language barriers, clinician training, and interdisciplinary coordination.

Specifically, the findings highlight the importance of structured communication tools—such as culturally adapted ACP resources and dignity therapy protocols—that are co-designed with families and tailored to the neurodevelopmental and cognitive limitations of pediatric patients. The implementation of models like PediSICP and written family feedback summaries shows promise in improving goal-concordant care, reducing parental anxiety, and fostering trust.

Additionally, ethically grounded communication practices—including early interdisciplinary alignment, transparent documentation, and inclusion of the primary care team—can mitigate fragmented messaging and prevent delayed referrals to PPC, especially in the NICU and neurology settings. Cultural humility must be seen not as a static skill but as an evolving clinical stance supported by ongoing education and systemic reform.

Future priorities should include the empirical validation of these communication innovations across diverse sociocultural contexts and neurological conditions. Ultimately, improving PPC communication means honoring the dignity, agency, and humanity of every child and family across all care settings.

## Figures and Tables

**Table 1:** Summary of the article selection process.

Stage	Description	Number of Articles
Initial search results	Articles retrieved from the database and manual searches, including additions from the authors’ library	170
Title and abstract screening	Excluded duplicates and articles unrelated to communication, culture, or pediatric palliative care	78
Full-text articles reviewed	Evaluated for relevance to PPC, culture, spirituality, religion, communication, and neurology	65
Final articles included in review	Articles included in thematic synthesis	43

**Table 2:** Core cultural dimensions shaping communication in pediatric palliative care.

Dimension	Cultural Variations	Communication Implications
Truth-telling and disclosure	Full disclosure vs. protective non-disclosure (e.g., Confucian, Middle Eastern values)	Clinicians should elicit family preferences and adapt the timing and content of prognosis discussions [22,23,24].
Decision-making dynamics	Individual autonomy vs. family-centered or hierarchical structures	Shared decision-making should be flexible, accommodating both collective and individual values [22,25].
Concepts of suffering	Suffering as redemptive, spiritual, or avoidable	Explore beliefs on suffering and align care goals sensitively, especially during end-of-life planning [26,27].
Language and interpretation	Limited health literacy, reliance on family interpreters	Use professional interpreters and assess understanding iteratively [5,21].
Spirituality and existential meaning	Diverse interpretations of illness and the afterlife	Encourage open-ended dialogue about faith, hope, and values [9,20].

**Table 3:** Unique communication challenges in neurologically ill children.

Challenge	Underlying Factors	Recommended Strategies
Prognostic uncertainty	Variable progression, lack of biomarkers, complex syndromes	Parallel planning; iterative, transparent communication [11,34].
Limited child participation	Cognitive/developmental impairments	Empower caregivers via structured models (e.g., PediSICP) [35].
Family overwhelm	Long-term caregiving stress, information overload	Gradual information delivery, documented summaries, interdisciplinary team support [36,37].
Fragmented communication	Multiple specialties, lack of coordination	Consistent involvement of the treating team; unified messaging in family meetings [4,25].
Ethical dilemmas in care decisions	Divergent team views; cultural-spiritual tension	Use of clinical ethics consultation; clear documentation of values and decisions [38].

**Table 4:** Promising communication innovations in cross-cultural PPC.

Innovation	Description	Applicability to Neuropalliative Care
Culturally adapted ACP tools (e.g., iCanCarePlan)	Co-designed resources with input from diverse communities	Adaptable with attention to cognitive/developmental constraints [39].
Family-based dignity therapy	Legacy-building and meaning-centered interventions	Effective in culturally collectivist settings; requires communication support [8].
WhatsApp or digital messaging	Asynchronous, informal clinician-family exchanges	Not yet validated in neuropalliative care; potential use for adolescents [40].
PediSICP communication model	Structured scripts and clinician prompts for goal-aligned conversations	Feasible in neuro-complex children with caregiver mediation [35].
Written summary with family feedback	Inviting family input on the documentation of PPC discussions	Enhances understanding and trust in families of neurologically ill children [36].
